# Configuration of biological wastewater treatment line and influent composition as the main factors driving bacterial community structure of activated sludge

**DOI:** 10.1007/s11274-013-1273-9

**Published:** 2013-02-09

**Authors:** Paulina Jaranowska, Agnieszka Cydzik-Kwiatkowska, Magdalena Zielińska

**Affiliations:** Department of Environmental Biotechnology, Faculty of Environmental Sciences, University of Warmia and Mazury in Olsztyn, Słoneczna 45G, Olsztyn, Poland

**Keywords:** Bacterial diversity, Bacterial community structure, WWTP design, DGGE, Canonical correspondence analysis

## Abstract

The structure of microbial consortia in wastewater treatment facilities is a resultant of environmental conditions created by the operational parameters of the purification process. In the research, activated sludge from nine Polish wastewater treatment plants (WWTPs) was investigated at a molecular level to determine the impact of the complexity of biological treatment line and the influent composition on the species structure and the diversity of bacterial consortia. The community fingerprints and technological data were subjected to the canonical correspondence and correlation analyses. The number of separated biological processes realized in the treatment line and the presence of industrial wastewater in the influent were the key factors determining the species structure of total and ammonia-oxidizing bacteria in biomass. The N_2_O-reducers community composition depended significantly on the design of the facility; the highest species richness of denitrifiers was noted in the WWTPs with separated denitrification tanks. The contribution of industrial streams to the inflow affected the diversity of total and denitrifying bacterial consortia and diminished the diversity of ammonia oxidizers. The obtained data are valuable for engineers since they revealed the main factors, including the design of wastewater treatment plant, influencing the microbial groups critical for the stability of purification processes.

**Electronic supplementary material:**

The online version of this article (doi:10.1007/s11274-013-1273-9) contains supplementary material, which is available to authorized users.

## Introduction

In wastewater treatment plants (WWTPs), the removal of nutrients should be maintained at a level ensuring the concentrations of pollutants in the effluent in accordance with the legal requirements. For this purpose, the alternating oxygen conditions must occur in the technological systems to enable the efficient nitrogen and phosphorus elimination from wastewater. In most WWTPs, in order to ensure the high efficiency of processes associated with the nutrient removal, anaerobic, anoxic and aerobic tanks are deliberately introduced into the technological line. In the WWTPs with a low flow rate, the biological treatment is performed only in aeration tanks, however, alternating oxic conditions may occur spontaneously as a result of limited oxygen diffusion into large flocs or thick biofilm.

The species structure of microbial communities in WWTPs decides the efficiency of the wastewater treatment. The knowledge about the ecology of the microbial communities responsible for the pollutant removal and the influence of the operational parameters and environmental variables on their structure and dynamics is significant for the optimization of the treatment systems (Gray et al. 2002; Molina-Muñoz et al. 2009). It was proved that the stability of wastewater treatment depends on the diversity of microbial groups responsible for the particular processes. The high species richness of microbial community ensures conservation of a given functionality and a quick recovery of the consortia after the stress conditions because of the functional redundancy and the alternative ways to use the flow of energy (Fernandez et al. 1999).

The threat posed by nitrogen and phosphorus to aquatic ecosystems requires the reduction of their load discharged into the environment. In most WWTPs, phosphorus is removed by chemical precipitation, so it is relatively easy to deal with its excessive load. Nitrogen is only removed as a result of the biological conversions, therefore it is necessary to ensure the high diversity and number of nitrifiers and denitrifiers in biomass. Nitrification is a two-phase process in which ammonium is oxidized to nitrite by ammonia-oxidizing bacteria (AOB) and then nitrite is oxidized to nitrate by nitrite-oxidizing bacteria (NOB). The path of nitrogen removal from wastewater is mostly via heterotrophic denitrification involving the reduction of nitrate and nitrite to gaseous products (NO, N_2_O, N_2_) under the conditions of the limited oxygen availability. Denitrifying bacteria belong to a broad variety of groups and encompass a wide range of physiological traits (Zumft 1997).

Since most of the microorganisms from the environmental samples cannot be cultivated, the monitoring of their diversity and number must involve molecular tools. In particular, polymerase chain reaction–denaturing gradient gel electrophoresis (PCR–DGGE) is reckoned as a rapid and reliable method for the relative comparison of the different bacterial communities (Muyzer et al. 1993). This fingerprinting method allows the direct measurement of the genetic biodiversity of a sample and coupled with sequencing and phylogenetic analyses can provide an overview of the microbial composition and diversity in the system (Sanz and Köchling 2007).

Many studies have focused on the analyzing the relations between the bacterial communities, especially nitrifiers, in biomass, and a type of wastewater (Limpiyakorn et al. 2011; Whang et al. 2009) in different bioreactors (Wan et al. 2011; Ye et al. 2011). The literature scarce the data on the influence of the full-scale wastewater treatment plant organization on the bacterial consortia in biomass. Therefore, the goal of the study was a robust statistical analysis of the dependence between the presence of the particular tanks and processes in the biological treatment line of WWTPs, the influent characteristics and the community structure of total, nitrifying and denitrifying bacteria in the activated sludge. The relations between the microbial assemblages and the technological data were investigated using the canonical correspondence analysis and correlation matrix.

## Materials and methods

### WWTPs

The WWTPs were chosen to obtain a broad spectrum of the possible technological solutions. We analyzed nine facilities operated in the activated sludge method that differed in the influent composition and the characteristics of the biological treatment line. The People Equivalent (PE) of analyzed WWTPs varied between 2,400 and 177,000. Five of the WWTPs, Tyrowo (TY), Jędrychówko (JE), Elbląg (EL), Nowe Gizewo (NG) and Olsztyn (OL) received industry and domestic wastewater. The remaining WWTPs, namely Gietrzwałd (GT), Jonkowo (JO), Rakowiec (RA) and Łyse (LY) treated domestic wastewater. The detailed characteristics of the facilities is given in Table 1. The technological results were obtained from WWTP operators, the influents and effluents from RA and GT were analyzed in our laboratory, according to APHA (1992).

**Table 1:** The treatment lines and characteristics of influents and effluents of investigated WWTPs

WWTP	Pre-settling	Configuration of bioreactors	Chemical P-precipitation	Q (m^3^/d)	Influent (g/m^3^)						Effluent (g/m^3^)				
					TSS	COD	BOD	TKN	P	COD/N	TSS	COD	BOD	N	P
LY	No	AxT, AT	No	360	nm	1,114±197	736±162	96±21	nm	12	nm	nm	nm	nm	nm
RA	Yes	AnT, AT	No	200	670±151	1,304±281	900±182	107±14	nm	12	27±8	88±22	16±8	95±22	nm
OL	Yes	Pre-DT, AnT, SNDT	Periodically	30,000	523±62	416±88	354±94	62±14	11±3	7	5±3	37±13	5±2	21±5	1±1
EL	Yes	AxT, AT	Yes	23,000	372±79	841±132	398±75	71±13	10±3	12	7±3	42±15	4±2	6±2	1±1
JE	No	AnT, SNDT	No	4,000	425±87	1,050±202	480±98	85±22	15±4	12	nm	nm	nm	nm	nm
TY	Yes	Pre-DT, AnT, AxT, AT	Periodically	6,660	601±182	1,520±375	1,005±301	110±16	19±11	14	9±5	64±17	8±5	15±4	1±1
GT	Yes	AT	Yes	129	nm	696±353	452±98	78±5	nm	9	nm	nm	nm	nm	nm
JO	No	Pre-DT, AT, Post-DT, UF	No	300	572±58	1,133±242	661±150	129±18	16±7	9	<0.2	28±6	2±1	3±1	6±1
NG	No	AnT, AxT, AT	Yes	6,300	312±97	1,019±172	408±109	74±15	11±4	14	3±2	26±7	3±2	11±2	1±1

*AT* aerobic tank, *AnT* anaerobic tank, *AxT* anoxic tank, *Pre*-*DT* pre-denitrification tank, *Post*-*DT* post-denitrification tank, *SNDT* simultaneous nitrification–denitrification tank, *UF* ultrafiltration, *Q* flow rate, *TSS* total suspended solids, *nm* not measured

### PCR–DGGE

The biomass from each WWTP was sampled twice from aerobic tanks in a period from January to March 2012 and was frozen in −20 °C prior to molecular analysis. DNA was extracted from approximately 400 mg of centrifuged sample using a FastDNA^®^ SPIN^®^Kit (Q-BIOgene, Canada). The working solutions with the DNA concentration of 50 ng/μL were prepared and the concentration of the DNA was measured spectrophotometrically using BioPhotometer (Eppendorf, Germany). The PCRs were performed in an Eppendorf^®^ Mastercycler Gradient (Eppendorf). The determination of the total bacteria diversity was based on 16S rDNA analysis with a primer set 341F/515R. The first-phase nitrifiers’ diversity was based on the analyses of *amoA* gene (301F/302R and amoA-1F/amoA-2R primer sets) that codes for the ammonium monooxygenase involved in the ammonium oxidation to nitrite. The denitrifying bacteria diversity was assessed based on the presence of *nosZ* gene (NosZ1/NosZ2 primer set), which codes for the nitrous oxide reductase responsible for the last step of denitrification (the gene is present in bacteria conducting full denitrification). The primer sequences are given in Table 2.

**Table 2:** Primers used in the study and DGGE conditions

Amplicon	Specificity	Primer set	PCR product length (bp)	Reference	Denaturant gradient; percentage of gel	Time and voltage of electrophoresis	Reference
16S rDNA	V3 region within the bacterial 16S rDNA	341F/515R	~230	Muyzer et al. (1993)	30–60 %; 6 %	4 h, 85 V	This study
*nosZ* gene	Nitrous oxide reducing bacteria	NosZ1/NosZ2	~500	Kloos et al. (2001), Throbäck et al. (2004)	40–70 %; 7 %	7 h, 120 V	This study
*amoA* gene	Ammonia-oxidizing bacteria	301F/302R	~700	Norton et al. (2002)	30–60 %; 6 %	4 h, 120 V	Cydzik-Kwiatkowska et al. (2012)
		amoA-1F/amoA-2R	~500	Rotthauwe et al. (1997)			

The PCR mixture contained 1.7 ng/μL of extracted DNA, 0.5 μM of each primer, 100 μM of deoxynucleoside triphosphate mixture (Promega, Madison, USA), 1.5 U of GoTaq^®^ DNA Polymerase (Promega), 6 μL of 10 × reaction buffer supplied with polymerase, 1.5 mM MgCl_2_ and sterile water to a final volume of 30 μL. The amplification of the *amoA* gene was performed as a nested-PCR (Cydzik-Kwiatkowska and Wojnowska-Baryła 2011). The thermal profile for the 16S rDNA amplification was: 94 °C for 5 min, 35 cycles of: denaturation at 94 °C for 45 s, annealing at 62 °C for 45 s, extension at 72 °C for 1 min, and a final elongation at 72 °C for 5 min. The thermal profile for the *nosZ* gene amplification was: 94 °C for 5 min, 6 cycles of touchdown PCR (denaturation 94 °C for 30 s, annealing for 1 min with an 1 °C for two cycles decrement at temperature 61 °C, extension at 72 °C for 1 min), followed by 25 cycles of 94 °C for 30 s, 58 °C for 1 min, 72 °C for 1 min and a final elongation for 10 min at 72 °C. The presence of the PCR products was confirmed by agarose electrophoresis. The amplified products were resolved on DGGE gels using a dCode System (Bio-Rad, USA), the electrophoresis conditions are presented in Table 2.

Denaturing gradient gel electrophoresis gels were stained with SYBR Gold (Invitrogen, USA) and digitalized using Kodak 1D 3.6 Image Analysis Software (Eastman Kodak Company, USA). The *amoA* amplicons that were clear and had a high intensity were excised from the DGGE gel, reamplified and sequenced in the Institute of Biochemistry and Biophysic, Polish Academy of Science (http://www.oligo.ibb.waw.pl). The nucleotide sequences were compared with the sequences in the GenBank using the BLASTn program (Altschul et al. 1997) and deposited in the GenBank under the accession No. JX546276-79. The sequences determined in this study were aligned and the genetic relationships were determined (the Maximum Likelihood method) using the MEGA5 software (Tamura et al. 2011).

### Calculation methods

In all tests, the significant effects were those with *p* value <0.05. For the calculations, the numerical values were assigned to express the number of separate biological processes realized in the biological treatment line of WWTPs and the presence of the tanks favoring denitrification (Table 3).

**Table 3:** Numerical values assigned to express the number of separate biological processes and the presence of the tanks favoring denitrification in WWTP treatment line

	Process/denitrification tanks	Numerical value
Process	Nitrification; carbon removal	1
	Nitrification; denitrification; carbon removal	2
	Phosphorus removal; nitrification; carbon removal	2
	Phosphorus removal; simultaneous nitrification/denitrification; carbon removal	2.5
	Phosphorus removal; nitrification; denitrification; carbon removal	3
Denitrification tanks	Lack of separate denitrification tank	0
	Pre-denitrification tank	0.5
	Post-denitrification tank	0.5
	Simultaneous nitrification/denitrification tank	0.75
	Separated denitrification tank	1

The canonical correspondence analysis (CCA) was performed on the relative DGGE band intensities with the Monte Carlo permutation testing (499 permutations). To the CCA analysis, next to metadata obtained by DGGE, the technological data such as the COD/N and BOD/COD ratios, TKN and COD in the influent, the presence (IN +) or absence (IN−) of industrial wastewater in the influent, the presence of the denitrification tanks (DT) and the number of processes designed to occur in the biological treatment line of WWTP (PR) were taken. The flow rate was removed from the analysis since it was strongly correlated (R = −0.98) with the COD concentration in the influent (ter Braak 1986). The values of DT and PR for each WWTP are presented in Table 4.

**Table 4:** Numerical values expressing denitrification tank presence and the number of processes realized in WWTPs taken for the CCA

WWTP	DT	PR
LY	1	2
RA	0	2
OL	1.25	2.5
EL	1	2
JE	0.75	2.5
TY	1.5	3
GT	0	1
JO	1	2
NG	1	3

The analyses were carried out using the CANOCO for Windows ver. 4.51 and CANODRAW (ter Braak, Smilauer, Biometris, Wageningen, The Netherlands). The correlations between the bacterial diversity and the technological parameters were analyzed using the correlation matrix in the Statistica 10.0 (StatSoft, USA). The statistical analysis assumed that the values from 0.9 to 1.0 point out to almost full correlation, very high correlation reaches the values from 0.7 to 0.9, strong—from 0.5 to 0.7, the medium correlation from 0.3 to 0.5, while weak from 0.1 to 0.3 (Stanisz 2000).

## Results

**Fig. 1:**
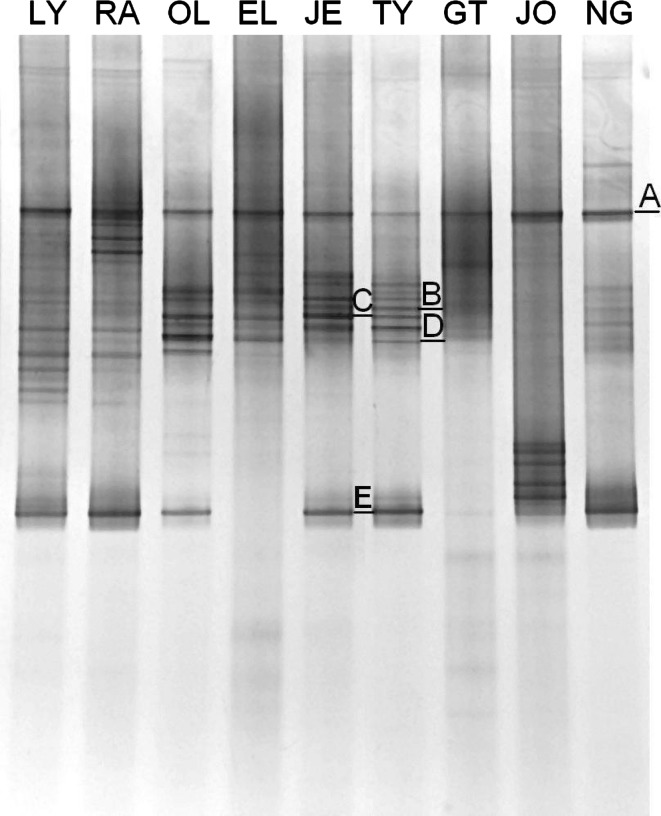
The DGGE analysis of PCR amplifications of the partial *amoA* gene. The abbreviations above each lane represent the WWTP the activated sludge samples were taken from. The bands that were sequenced are marked with capital letters

**Fig. 2:**
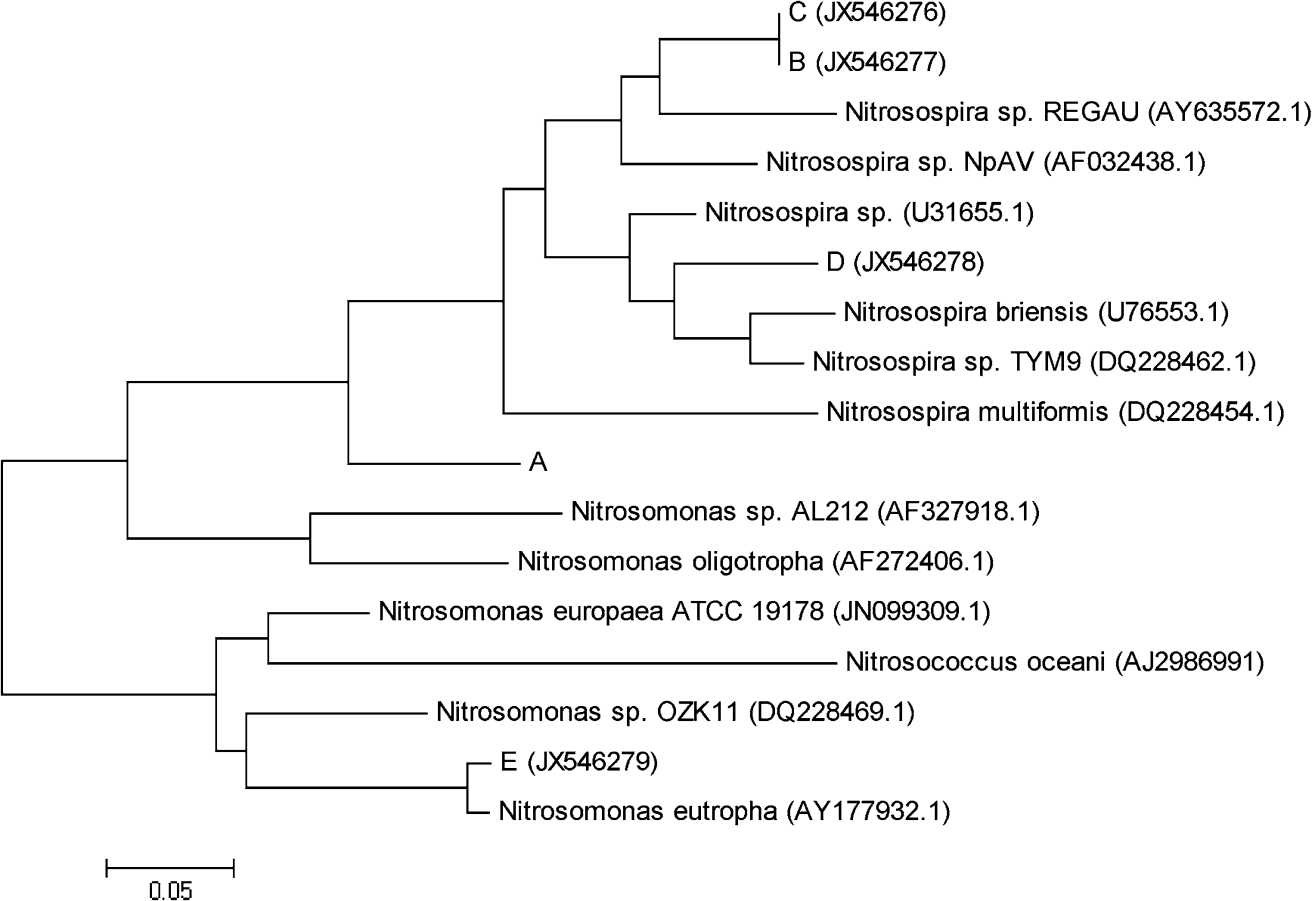
Phylogenetic tree showing the relationships of the partial *amoA* gene sequences to reference the sequences from GeneBank database (accession numbers given in parentheses). The sequence of band A is given in Supplementary material. The evolutionary history was inferred by using the Maximum Likelihood method based on the Tamura-Nei model (Tamura and Nei 1993). Initial tree(s) for the heuristic search were obtained automatically as follows. When the number of common sites was <100 or less than one fourth of the total number of sites, the maximum parsimony method was used; otherwise the BIONJ method with MCL distance matrix was applied. The tree is drawn to scale, with branch lengths measured in the number of substitutions per site. Evolutionary analyses were conducted in the MEGA5 (Tamura et al. 2011)

In the present research, to analyze the bacterial assemblages, the DNA isolated from the biomass was amplified using the specific primer sets and the obtained products were separated in DGGE. In general, a higher number of bands indicates greater diversity of the analyzed microbial consortia. The DGGE separation of the *amoA* PCR products is presented in Fig. 1, the DGGE separations of the 16S rDNA and *nosZ* gene are presented as supplementary materials (Fig. SM-1, Fig. SM-2). The sequencing of the *amoA* bands and the phylogenetic analysis (Fig. 2) showed that most of the sequenced bands (A, B, C, D) were related to the *Nitrosospira* sp. The band E was closely related to *Nitrosomonas eutropha*. This band was in all the plants except EL, we also detected only a weak band E in GT.

The canonical correspondence analysis is designed for relating the species composition of communities to their environment and can provide an insight into the impact of wastewater treatment plant design and the operational parameters on the bacterial assemblages. The data analysis can be used in an explanatory way and it leads to an ordination diagram of samples, species and environmental variables, which optimally displays how the community composition varies with the environment. When used in a confirmatory way, it leads to statistical tests of the effects of particular environmental variables on the community composition taking into account the effect of other variables (ter Braak and Smilauer 2002).

**Fig. 3:**
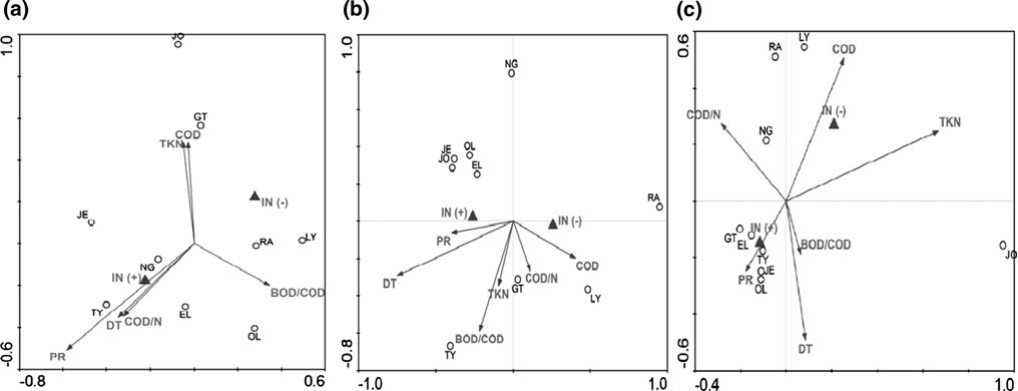
The CCA of **a** total **b** N_2_O-reducing and **c** ammonia-oxidizing bacteria communities; the discrete (*triangle*) (IN ± = the presence/absence of industrial wastewater in the influent) and the continuous variables (*right arrow*) (the COD/N and BOD/COD ratios of the influent, COD and TKN in the influent, DT—presence of the denitrification tanks, PR—the number of the processes realized in the biological treatment line of WWTP)

In Fig. 3a, the points represented the total bacterial consortia in different WWTPs while the environmental variables were represented by arrows. The bacterial community structure differed between the WWTPs and no separate clusters were observed in the diagram. The first axis of a biplot explained the 24 % of the species-environment relation, while the summary variation explained by the two-dimensional diagram was 42 %. The first and the second eigenvalues equaled 0.31 and 0.23, respectively. The species-environment correlations of the first two axes were very high (above 99 %) showing that the measured environmental variables were sufficient to explain the major variations among the analyzed WWTPs. The correlation coefficients showed that the first axis was a presence or an absence of industrial wastewater in the WWTP influent (R = ± 0.71). The discrete variable IN had the highest power to explain the patterns in the species data and the significance of the explanatory effect was statistically important (Monte Carlo permutation test, F = 1.48, *p* = 0.05). The correlations of the second axis showed a contrast between the WWTPs with the different number of processes realized in the treatment line (R = 0.50) and different TKN and COD concentrations in the influent (R = 0.48). The length of an arrow representing an environmental variable is a measure of how much the total bacteria community structure differs along that environmental variable. Since the PR arrow was the longest one, the number of processes realized in the WWTPs was the most important continuous environmental variable influencing the total bacteria community structure in activated sludge from all analyzed.

From Fig. 3b it can be concluded that the points representing the N_2_O-reducing bacteria communities in JE, JO, OL and EL were similar and grouped together in the diagram. The first and the second axes explained 36 and 20 % of the species-environment relation with the respective eigenvalues of 0.41 and 0.24. The species-environment correlations for the first axis was 0.98 while for the second one it equaled 0.99. The correlation coefficients showed that the first axis is DT in the WWTP treatment line (R = −0.73). This environmental continuous variable had the highest power to explain the DGGE patterns of denitrifiers, and the significance of the explanatory effect was statistically important (Monte Carlo permutation test, F = 1.97, *p* = 0.02). The correlations of the second axis showed that the BOD/COD ratio of the influent was the second environmental variable influencing mostly the N_2_O-reducing bacteria communities in activated sludge from the analyzed WWTPs (R = −0.58).

The CCA analysis of the DGGE patterns characterizing the AOB communities showed that the most similar assemblages were in GT, EL, TY, JE and OL (Fig. 3c). The first axis of a biplot explained the 30 % of the species-environment relation, while the summary variation explained by the two-dimensional diagram was high and equaled 53 %. The first and the second eigenvalues equaled 0.57 and 0.45, respectively. The species-environment correlations of the first two axes were above 99 %. The correlation coefficients showed that the first axis is concentration of TKN in the influent (R = 0.67) and that this factor was the most important continuous environmental variable. The second axis was the discrete variable namely the presence/absence of industrial wastewater in the influent (IN; R = ± 0.65). The significance of the explanatory effect of IN was statistically important (Monte Carlo permutation test, F = 1.82, *p* < 0.00).

**Fig. 4:**
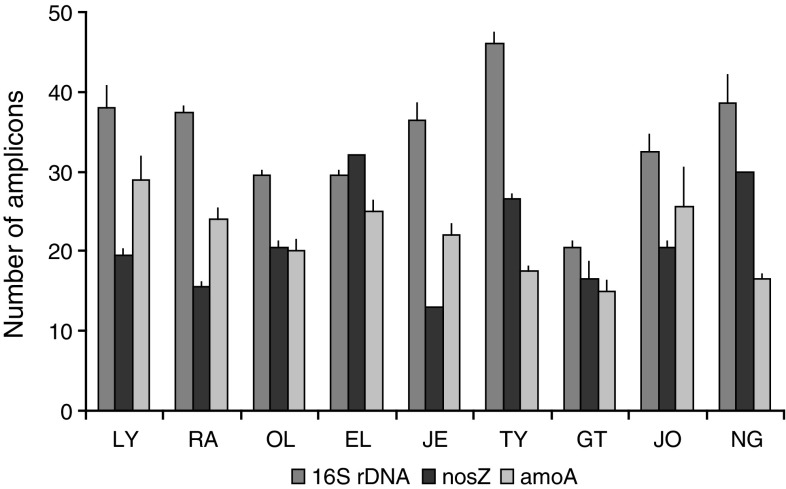
The average number of 16S rDNA, *nosZ*, *amoA* bands in the DGGE patterns characterizing activated sludge communities from analyzed WWTPs; the averages of two different measurements, standard deviations are given

Fig. 4 presents the microbial diversity expressed as the average number of amplicons in the DGGE patterns. The number of 16S rDNA bands varied depending on the WWTP. The highest number of amplicons (47) was in TY, the WWTP with a technological line ensuring the removal of carbon, nitrogen and phosphorus with the respective efficiency of 96, 86, 95 %. The lowest number of 16S rDNA amplicons was obtained in GT. Three facilities (JE, RA, GT) operated without the separated denitrification tanks had the lowest diversity of denitrifiers (13, 16 and 15 *nosZ* bands, respectively) in activated sludge. The highest diversity of denitrifiers (from 26 to 32 *nosZ* bands) was obtained in activated sludge from EL, TY and NG. The highest diversity of AOB (ca. 27 bands) was noted for the biomass from JO and LY. The lowest ammonia-oxidizers diversity characterized activated sludge from GT.

The correlation matrix was constructed to find the dependences between the diversity of particular groups of bacteria (total, N_2_O-reducing and ammonia-oxidizing) and the investigated WWTP design and the influent composition. It was observed that the total bacteria diversity was strongly correlated with the COD/N ratio (R = 0.84) and the number of processes realized in the facility (R = 0.79). We also obtained a positive strong correlation (R = 0.50) between the 16S rDNA band number and the presence of denitrification tank. As to the facility design, the presence of anoxic tanks had the greatest impact on the diversity of N_2_O-utilizing denitrifiers (R = 0.57). Analyzing the wastewater characteristics, both the presence of the industrial stream and the high COD/N ratio in the WWTP influent influenced the species richness of denitrifiers (R = 0.51 and R = 0.48, respectively). The AOB diversity was weakly positively correlated with the TKN concentration in the influent (R = 0.25) and negatively influenced by the presence of industrial wastewater in the influent (R = −0.25).

## Discussion

In the research, activated sludge from nine WWTPs was investigated to determine the influence of the complexity of the biological treatment line and the influent characteristics on the structure and diversity of microbial consortia. For the statistical analysis, the CCA and correlation matrix were applied.

The species composition of the total bacterial communities in activated sludge was influenced by both the design of the treatment line and the wastewater characteristics. The CCA analysis showed that the number of processes realized in the WWTPs was the most important of all continuous environmental variables deciding about the structure of total bacteria assemblages. The complexity of the treatment line positively affected also the overall diversity of bacteria in the biomass. The highest number of different bacterial species was noted for TY with a complex, many-stage technological line. Reversely, the lowest number of 16S rDNA bands characterized activated sludge community in GT, the smallest of all analyzed WWTPs (PE = 2,405) with the simplest biological treatment line consisting only of the aeration tank. It can be concluded that the presence of many tanks with the different oxic conditions in the treatment line favors the growth of multispecies microbial consortia thus promoting the stability of the purification processes. As to the wastewater composition, the statistically significant influence of the industrial wastewater presence on both the structure and the diversity of the total bacteria communities was proven. Since the origin of industrial wastewater in the analyzed treatment facilities was broad, it was difficult to claim which components of the sewage promoted the biodiversity. Our results, however, pointed out that the presence of the industrial stream favored species richness of total bacteria in activated sludge due to the accessibility of a broader range of substrates as compared to that present in typical domestic wastewater.

The WWTPs are one of the major sources of nitrogen oxides, especially N_2_O. Many parameters such as a substrate concentration, a C/N ratio, a type of carbon source, a nitrite accumulation and an NO concentration influence the N_2_O production (Adouani et al. 2010). The knowledge about the links between the wastewater treatment line design and the structure of N_2_O-utilizing bacteria communities in the biomass would allow engineers to apply the purification strategy favoring the effective reduction of the greenhouse gas emission. The research proved that the number of separated denitrification tanks in the WWTPs was the major factor deciding about the species composition and the diversity of the N_2_O-reducing microorganisms in activated sludge. In three facilities (JE, RA, GT) operated without the separated denitrification tanks, the lowest species richness of denitrifiers in the biomass was noted. In JE, the tank with the simultaneous nitrification/denitrification was exploited meaning that nitrifiers and denitrifiers co-existed in activated sludge, however, the alternating oxic conditions did not favor the full denitrification to N_2_ since the activity of bacteria that possess the *nosZ* genes is inhibited in the presence of even low oxygen concentrations (Hochstein et al. 1984). For comparison, in OL, nitrogen removal was realized by SND in a carrousel-type bioreactor, however there was an additional tank for pre-denitrification favoring the growth of more diverse assemblage of the N_2_O-utilizing denitrifiers. The highest diversity of denitrifiers was obtained in activated sludge from EL, TY and NG. These WWTPs were characterized by both the presence of separate anoxic tanks and the highest ratio of COD/N (12–14) that was also proven to positively influence the species richness of investigated bacteria (R = 0.48).

The microorganisms capable of denitrification belong to a broad variety of groups and encompass a wide range of the physiological traits. Most denitrifiers are the aerobic heterotrophic organisms that transfer redox equivalents from the oxidation of a carbon source to N oxides under the anaerobic conditions. The ability to reduce N_2_O by the nitrifying and phosphorus accumulating bacteria is also documented (Zumft 1997). This can explain the tendency observed in the current research that more processes realized separately in the treatment line favored the diversity of N_2_O-reducers.

Siripong and Rittman (2007) investigated the AOB communities in activated sludge in seven typical single-stage municipal plants. Among the analyzed factors (flow rate, influent and effluent BOD and TKN, effluent ammonia, nitrite and nitrate, pH, and sewage temperature), only the seasonal temperature variations seemed to change the nitrifying community, especially the balance between *Nitrosospira* sp. and *Nitrosomonas* sp., although both genera coexisted in winter and summer samples. Lydmark et al. (2007) showed that ammonium concentration was an important structuring factor for an AOB community. In the present research, the CCA proved that the main variable influencing the AOB consortia in activated sludge was the presence of the industrial stream in the WWTPs influent. In fact, the IN variable was the only one that had a statistically significant impact on the species composition of the AOB. These results are well depicted in Fig. 3c. The DGGE patterns characterizing activated sludge from GT, EL, TY, JE and OL facilities with the lower values of COD and TKN in the influent (except from TY) and the presence of industrial influent (except from GT) were grouped together in a separate cluster.

The coexistence of various nitrifiers in WWTP is an evidence of a functional redundancy, a feature that may help in the maintaining the stability of the system for nitrification. Wang et al. (2010) investigated the communities of microorganisms in activated sludge of eight wastewater treatment systems and suggested the negative impact of the presence of industrial wastewater in the influent on the nitrifiers’ diversity. Our observations pointed out to a weak negative correlation between the industrial wastewater presence and the species richness of AOB (R = −0.25), nevertheless, the highest diversity of these bacteria was noted in biomass from JO and LY that received only domestic wastewater. The lowest AOB diversity characterized activated sludge from GT, the WWTP with the simplest technological line consisting of only an aeration tank. It can be concluded that in this system, under a stable oxygen concentration, the microorganisms underwent a strong selective pressure favoring the growth of only a few best adapted species. Whang et al. (2009) evaluated the nitrifying community and the nitrification performance of the full-scale municipal (20 mg N/L) and swine (220 mg N/L) WWTPs. Authors observed dissimilar nitrifying populations prevailing in these two plants and related this fact to different input nitrogen concentrations. In our research, in the analyzed range of the influent TKN (62 ± 14–129 ± 18 mg/L), only a weak correlation between this parameter and the AOB diversity was proven.

In general, the members of the *Nitrosospira* spp. or/and the *Nitrosomonas oligotropha* clusters are the dominant AOB in the ammonia-low environments, whereas the members of the *N. europaea*–*Nitrosococcus mobilis* cluster comprise the majority of AOB in the ammonia-rich environments (Limpiyakorn et al. 2005). The sequencing of the chosen *amoA* bands and the phylogenetic analysis showed that the AOB in the analyzed activated sludge samples were related to both *Nitrosomonas* sp. and *Nitrosospira* sp. (Fig. 2). An interesting observation can be made on band E, obtained from *N. eutropha*. This species was present in all the WWTPs except from EL, in GT only a weak band was observed. *N. eutropha* is commonly found in strongly eutrophic environments such as the municipal and industrial sewage disposal systems. This microorganism has a high tolerance for both elevated ammonia concentrations and the fluctuating conditions (especially oxic/anoxic cycles) (Koops et al. 1991; Stein et al. 2007). The low number of *N.* *eutropha* in GT (DGGE is a semi-quantitative technique so such a statement is justified) can be explained by the simplicity of the biological treatment line in this WWTP—it consisted of only an aeration tank. The stable oxic conditions and a lack of environmental fluctuations did not favor the growth of the analyzed species. The EL WWTP, on the other hand, received wastewater from a wood industry that did not use water as an input for the manufacturing processes but generated several wastewater streams after washing/cleaning procedures. In the production of wood-based floors and wood-laminates, small volumes of highly polluted wastewaters with the high contents of formaldehyde and COD are generated (e.g. cleaning of machines that are used to apply urea–formaldehyde resins onto wood-fiber boards) (Kaczala et al. 2010). Wastewater with formaldehyde could have negatively influenced the AOB community in EL, especially *N. eutropha*, since its genome lacks genes for urease metabolism (Stein et al. 2007).

## Electronic supplementary material

Below is the link to the electronic supplementary material.

Supplementary material 1 (DOCX 145 kb)

## References

[CR1] Adouani N, Lendormi T, Limousy L, Sire O (2010). Effect of the carbon source on N_2_O emissions during biological denitrification. Resour Conserv Recyl.

[CR2] Altschul SF, Madden TL, Schaffer AA, Zhang J, Zhang Z, Miller W, Lipman DJ (1997). Gapped BLAST and PSI-BLAST: a new generation of protein database search programs. Nucleic Acids Res.

[CR3] APHA (1992). Standard Methods for the Examination of Water and Wastewater.

[CR4] Cydzik-Kwiatkowska A, Wojnowska-Baryła I (2011). Nitrifying granules cultivation in a sequencing batch reactor at a low organics-to-total nitrogen ratio in wastewater. Folia Microbiol.

[CR5] Cydzik-Kwiatkowska A, Zielińska M, Wojnowska-Baryła I (2012). Impact of operational parameters on bacterial community in a full-scale municipal wastewater treatment plant. Pol J Microbiol.

[CR6] Fernandez A, Huang SY, Seston S, Xing J, Hickey R, Criddle C, Tiedje J (1999). How stable is stable? Function versus community composition. Appl Environ Microbiol.

[CR7] Gray NG, Miskin IP, Kornilova O, Curtis TP, Head IM (2002). Occurrence and activity of Archaea in aerated activated sludge wastewater treatment plants. Environ Microbiol.

[CR8] Hochstein IL, Betlach M, Kritikos G (1984). The effect of oxygen on denitrification during steady-state growth of *Paracoccus halodenitrificans*. Arch Microbiol.

[CR9] Kaczala F, Marques M, Hogland W (2010). Biotreatability of wastewater generated during machinery washing in a wood-based industry: COD, formaldehyde and nitrogen removal. Bioresour Technol.

[CR10] Kloos K, Mergel A, Rösch C, Bothe H (2001). Denitrification within the genus *Azospirillum* and other associative bacteria. Aust J Plant Physiol.

[CR11] Koops H-P, Böttcher B, Möller U, Pommerening-Röser A, Stehr G (1991). Classification of eight new species of ammonia-oxidizing bacteria: *Nitrosomonas communis* sp. nov., *Nitrosomonas ureae* sp. nov., *Nitrosomonas aestuarii* sp. nov., *Nitrosomonas marina* sp. nov., *Nitrosomonas nitrosa* sp. nov., *Nitrosomonas eutropha* sp. nov., *Nitrosomonas oligotropha* sp. nov. J Gen Microbiol.

[CR12] Limpiyakorn T, Shinohara Y, Kurisu F, Yagi O (2005). Communities of ammonia-oxidizing bacteria in activated sludge of various sewage treatment plants in Tokyo. FEMS Microbiol Ecol.

[CR13] Limpiyakorn T, Sonthiphand P, Rongsayamanont C, Polprasert C (2011). Abundance of *amoA* genes of ammonia-oxidizing archaea and bacteria in activated sludge of full-scale wastewater treatment plants. Bioresour Technol.

[CR14] Lydmark P, Almstrand R, Samuelsson K, Mattsson A, Sörensson F, Lindgren PE, Hermansson M (2007). Effects of environmental conditions on the nitrifying population dynamics in a pilot wastewater treatment plant. Environ Microbiol.

[CR15] Molina-Muñoz M, Poyatos JM, Sánchez-Peinado MM, Hontoria E, González-López J, Rodelas B (2009). Microbial community structure and dynamics in a pilot-scale submerged membrane bioreactor aerobically treating domestic wastewater under real operation conditions. Sci Total Environ.

[CR16] Muyzer G, de Waal EC, Uitterlinden AG (1993). Profiling of complex microbial populations by denaturing gradient gel electrophoresis analysis of polymerase chain reaction-amplified genes coding for 16S rRNA. Appl Environ Microbiol.

[CR17] Norton JM, Alzerreca JJ, Suwa J, Klotz MG (2002). Diversity of ammonia monooxygenase operon in autotrophic ammonia-oxidizing bacteria. Arch Microbiol.

[CR18] Rotthauwe JH, Witzel KP, Liesack W (1997). The ammonia monooxygenase structural gene amoA as a functional marker: molecular fine scale analysis of natural ammonia-oxidizing populations. Appl Environ Microbiol.

[CR19] Sanz JL, Köchling T (2007). Molecular biology techniques used in wastewater treatment: an overview. Process Biochem.

[CR20] Siripong S, Rittman BE (2007). Diversity study of nitrifying bacteria in full-scale municipal wastewater treatment plants. Water Res.

[CR21] Stanisz A (2000). Podstawy Statystyki dla Prowadzących Badania Naukowe. Odcinek 21: Analiza korelacji. Med Praktyczna.

[CR22] Stein LY, Arp DJ, Berube PM, Hauser L, Jetten MS, Klotz MG, Larimer FW, Norton JM, Op den Camp HJ, Shin M, Wei X (2007). Whole-genome analysis of the ammonia-oxidizing bacterium, *Nitrosomonas eutropha* C91: implications for niche adaptation. Environ Microbiol.

[CR23] Tamura K, Nei M (1993). Estimation of the number of nucleotide substitutions in the control region of mitochondrial DNA in humans and chimpanzees. Mol Biol Evol.

[CR24] Tamura K, Peterson D, Peterson N, Stecher G, Nei M, Kumar S (2011). MEGA5: molecular evolutionary genetics analysis using maximum likelihood, evolutionary distance, and maximum parsimony methods. Mol Biol Evol.

[CR25] ter Braak CJF (1986). Canonical correspondence analysis: a new eigenvector technique for multivariate direct gradient analysis. Ecology.

[CR26] ter Braak CJF, Smilauer P (2002). CANOCO Reference manual and CanoDraw for Windows user’s guide: software for the canonical community ordination (version 4.5). Microcomputer Power.

[CR27] Throbäck IN, Enwall K, Jarvis Ä, Hallin S (2004). Reassessing PCR primers targeting *nirS*, *nirK* and *nosZ* genes for community surveys of denitrifying bacteria with DGGE. FEMS Microbiol Ecol.

[CR28] Wan CY, Wever HD, Diels L, Thoeye C, Liang JB, Huang LN (2011). Biodiversity and population dynamics of microorganisms in a full-scale membrane bioreactor for municipal wastewater treatment. Water Res.

[CR29] Wang X, Wen X, Criddle C, Wells G, Zhang J, Zhao Y (2010). Community analysis of ammonia-oxidizing bacteria in activated sludge of eight wastewater treatment systems. J Environ Sci.

[CR30] Whang LM, Chien ICh, Yuan SL, Wu YJ (2009). Nitrifying community structure and nitrification performance of full-scale municipal and swine wastewater treatment plants. Chemosphere.

[CR31] Ye L, Shao MF, Zhang T, Tong AHY, Lok S (2011). Analysis of the bacterial community in a laboratory-cale nitrification reactor and a wastewater treatment plant by 454-pyrosequencing. Water Res.

[CR32] Zumft WG (1997). Cell biology and molecular basis of denitrification. Microbiol Mol Biol R.

