# Supplementary material for: Configuration of biological wastewater treatment line and influent composition as the main factors driving bacterial community structure of activated sludge
Source: World J Microbiol Biotechnol. 2013 Feb 9;29(7):1145–53. doi: 10.1007/s11274-013-1273-9 (PMC3683147; doi:10.1007/s11274-013-1273-9)
Supplement: Supplementary file 1 — Supplementary material 1 (DOCX 145 kb) [file 11274_2013_1273_MOESM1_ESM.docx]

**Configuration of biological wastewater treatment line and influent composition as the main factors driving bacterial community structure**

Paulina Jaranowska, Agnieszka Cydzik-Kwiatkowska*, Magdalena Zielińska

Department of Environmental Biotechnology, Faculty of Environmental Sciences, University of Warmia and Mazury in Olsztyn, Słoneczna 45G, Olsztyn, Poland

* Corresponding author: [agnieszka.cydzik@uwm.edu.pl](mailto:agnieszka.cydzik@uwm.edu.pl), tel. +48 89 5234194, fax +48 89 5234131

**
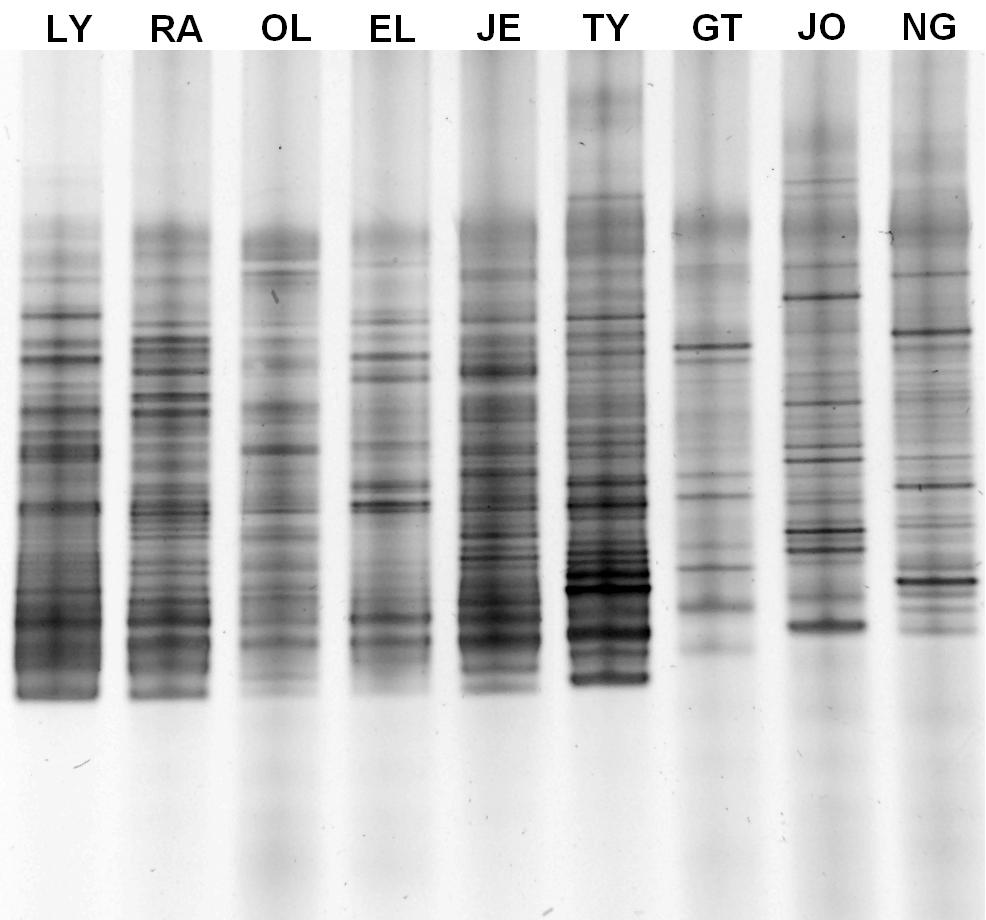
**

**Fig. SM-1** DGGE analysis of PCR amplifications of partial 16S rDNA gene. The abbreviations above each lane represent WWTP the activated sludge samples were taken from

**
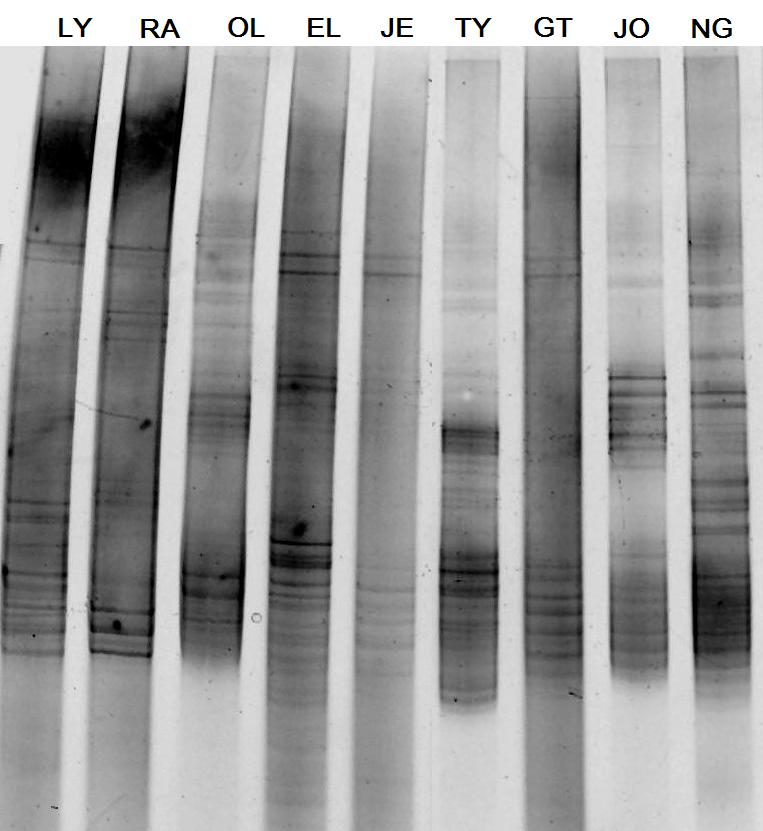
**

**Fig. SM-2** DGGE analysis of PCR amplifications of partial *nosZ* gene. The abbreviations above each lane represent WWTP the activated sludge samples were taken from

The sequence of DGGE band A

>band A [organism=Uncultured bacterium] isolate DGGE gel band A ammonia monooxygenase (amoA) gene, partial cds

ACAGGAAACCGGTGTAGTCAGAAAGTGACAGCAACACGCCTTCTACCACCAGTGGCAGATGGGTTGGTCCGAAGATCGGCCAGTTGCCAGGGTAGAACAGCAGTCCGAATGCGCC
